# Drug interactions in a sample of inpatients diagnosed with cannabis use disorder

**DOI:** 10.1007/s00702-025-02884-5

**Published:** 2025-01-23

**Authors:** Martin Schulze Westhoff, Christina Massarou, Stefan Bleich, Johannes Heck, Konstantin Fritz Jendretzky, Alexander Glahn, Sebastian Schröder

**Affiliations:** 1 Department of Psychiatry, Social Psychiatry and Psychotherapy, Hannover Medical School, Carl-Neuberg-Str. 1, 30625 Hannover, Germany; 2 Institute for Clinical Pharmacology, Hannover Medical School, Hannover, Germany; 3 Department of Neurology, Hannover Medical School, Hannover, Germany

**Keywords:** Cannabis, Cannabis-drug interactions, Drug Safety, Cannabis use disorder, Drug-drug interactions

## Abstract

The majority of patients with cannabis use disorder (CUD) regularly take medication. Cannabinoids influence metabolism of some commonly prescribed drugs. However, little is known about the characteristics and frequency of potential cannabis-drug (CDIs) and drug-drug interactions (DDIs) in patients with CUD. Therefore, our study aimed to determine the prevalence and characteristics of drug interactions in patients with CUD during inpatient treatment on an addiction-specific ward over a six-year-period. To this aim, medication charts were analyzed and screened for potential CDIs and DDIs. Herein, the drugs.com classification for potential CDIs and UpToDate Lexicomp program for potential DDIs were utilized. The study cohort consisted of 301 patient cases, predominantly male (85.0%), with a median age of 37 years. 89.4% (269/301) of all cases involved were taking at least one drug that could potentially interact with cannabis. Levomethadone, buprenorphine and morphine were the most common drugs involved in potentially serious CDIs. In addition, 196 DDIs were identified, of which 25.5% were classified as ‘avoid combination’ and 74.5% as ‘consider therapy modification’. Hereby, combinations of levomethadone with other psychotropic drugs most frequently accounted for potentially severe and mild DDIs. The results of our study indicate that especially patients diagnosed with CUD also receiving opioid substitution therapy are at risk for potential drug interactions. Therefore, a clinical monitoring of vigilance and respiratory function should be applied during inpatient treatment. Routine use of interaction check tools in patients diagnosed with CUD should also be considered by healthcare providers. In addition, therapeutic drug monitoring (TDM) should be used to increase medication safety in this patient population.

**Supplementary Information:**

The online version contains supplementary material available at 10.1007/s00702-025-02884-5.

## Introduction

Cannabis is the third most used controlled substance worldwide, following alcohol and tobacco (Connor et al. 2021). In 2018, approximately 192 million individuals worldwide reported cannabis consumption within the past year (Connor et al. 2021). Although global data on cannabis use disorder (CUD) is limited, it is estimated that about 10% of all consumers meet diagnostic critria (Connor et al. 2021; Gendy et al. 2023). According to the Diagnostic and Statistical Manual of Mental Disorders 5 (DSM-5), CUD can be diagnosed depending on the presence of at least two out of eleven symptoms such as craving, ongoing use despite physical harm or withdrawal symptoms when abstaining from consumption (Fink et al. 2022).

Additionally, many countries are moving towards legalizing the purchase and use of cannabis (Assanangkornchai et al. 2023; Walker et al. 2023). These developments appear to be associated with an increase in the prevalence of CUD, although there is still insufficient data on this at population level (Choo et al. 2022; Hasin et al. 2023). In view of this, cannabis consumption is perceived as less harmful, especially among younger adults (Urits et al. 2020; Chung et al. 2022).

Otherwise, people who regularly use cannabis are more likely to suffer from diseases of the cardiovascular system, lungs and liver (Bonnet and Scherbaum 2010). Moreover, various psychiatric disorders are common in patients with CUD (Gobbi et al. 2019; Kondev et al. 2021). This affects not only posttraumatic stress disorder or major depression, but also schizophrenic psychosis where cannabis use is characterized as an independent risk factor (Vaucher et al. 2018). The results of a Danish register-based cohort study recently showed that around a fifth of all schizophrenia cases in the group of young men could be avoided through the prevention of CUD (Hjorthoj et al. 2023). In addition, a meta-analysis by Marconi et al. utilizing a logistic regression model revealed an odds ratio of 3.9 for heavy cannabis users compared to non-users regarding the risk of schizophrenia (Marconi et al. 2016).

The prevalence of multiple diagnoses often requires drug prescriptions in patients with CUD (Connor et al. 2021). At the same time, there is growing interest in and opportunity for the medical use (e.g. for the treatment of chronic pain, epilepsy or multiple sclerosis) of cannabis and its active substances, leading to increased regular use (Legare et al. 2022).

Cannabis contains more than 100 cannabinoids, including delta-9-tetrahydrocannabinol (THC) and cannabidiol (CBD) (Solmi et al. 2023). Several of these components can inhibit or induce the activity of hepatic enzymes crucial for drug metabolism like cytochrome P450 (CYP) isoenzymes or UDP-glucuronosyltransferase (UGT) (Bansal et al. 2023). Therefore, potential pharmacokinetic interactions with concomitantly taken drugs should be considered (Lucas et al. 2018; Bansal et al. 2023). In addition, (recreational or medical) cannabis use carries the risk of pharmacodynamic interactions with other drugs, which may result in worsening adverse drug reactions (ADRs) like sedation or cognitive impairment (Lucas et al. 2018). In addition, strong CYP1A2 inducing effects of cannabis when consumed by smoking should be considered, especially under therapy with drugs like olanzapine, clozapine or theophylline (Anderson and Chan 2016).

Further insights into prevalence and etiology of potential cannabis-drug interactions (CDIs) are therefore urgently needed. To date, research has been limited to in-vitro studies on the influence of cannabinoids on CYP isoenzymes and to case reports on possible clinical consequences of pharmacokinetic interactions between cannabinoids and individual drugs (Geffrey et al. 2015; Grayson et al. 2018; Doohan et al. 2021). To the best of our knowledge, no study has investigated pattern of medication safety in patients diagnosed with CUD.

To this aim, the present study retrospectively analyzed potential drug-drug interactions (DDIs) and CDIs in inpatients diagnosed with CUD during treatment on an addiction-specific ward at a university hospital in Germany over a six-year period.

## Methods

### Ethics approval

The study was approved by the Ethics Committee of Hannover Medical School (No. 10764_BO_K_2023) and adhered to the Declaration of Helsinki and its later amendments.

### Study setting

**Fig. 1:**
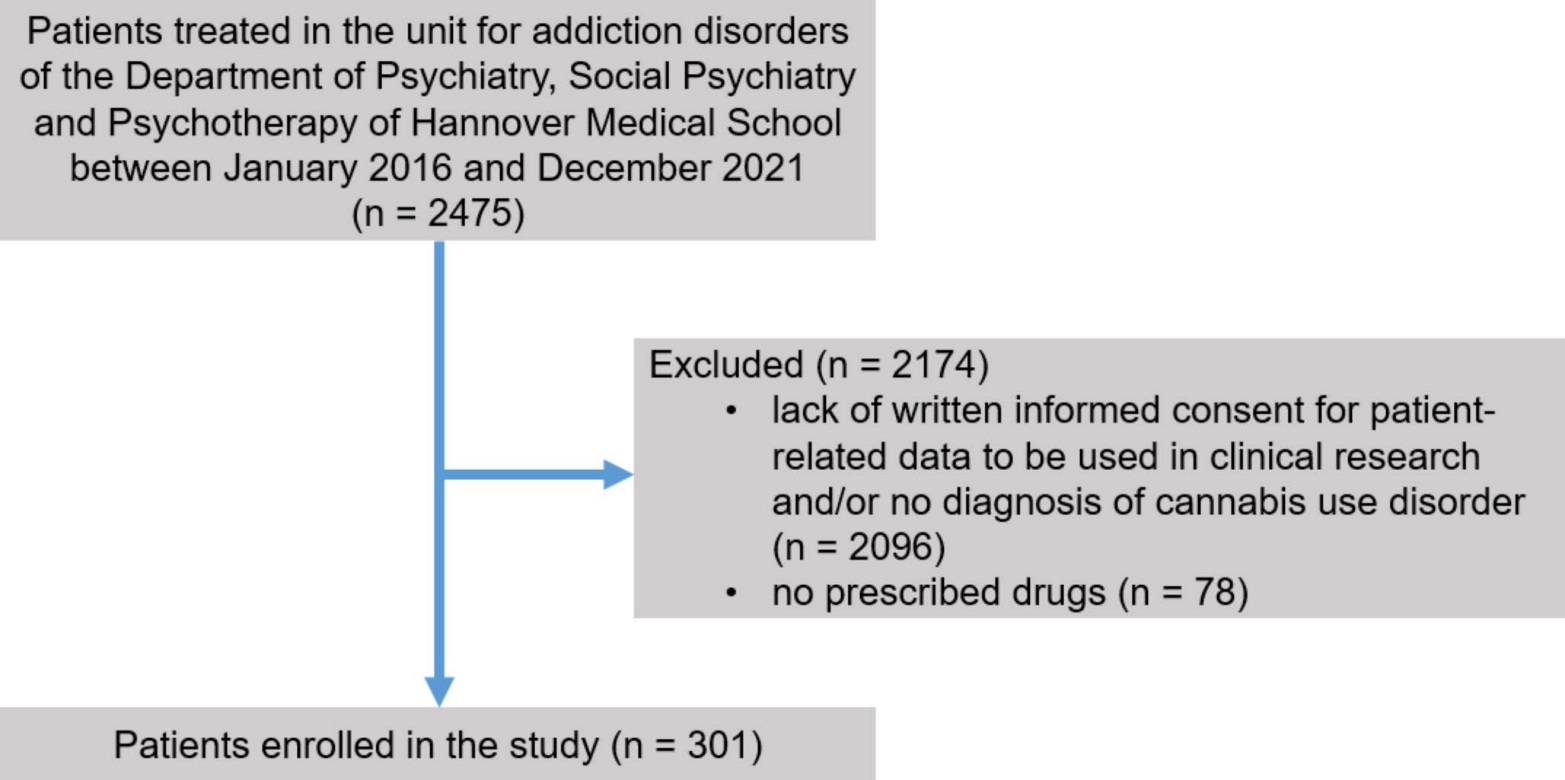
Flow of participants

The study was conducted as a retrospective cohort study. Patients were included in the study, if (i) they were treated on the addiction-specific ward of the Department of Psychiatry, Social Psychiatry and Psychotherapy of Hannover Medical School between January 2016 and De-cember 2021, (ii) they were diagnosed with CUD according to the International Classification of Diseases-10 (ICD-10) criteria and (iii) they or their legal representative had provided written informed consent that patient-related data be used for clinical research (Fig. 1). Hannover Medical School is a large university hospital and tertiary care referral center in northern Germany. The addiction-specific ward is a 12-bed facility specialized in the treatment and care of patients with substance use disorders. Patients were admitted for qualified withdrawal treatment from either one or more of the following substances: alcohol, cocaine, sedatives, amphetamines and opioids. In contrast to the other substances listed above, complete detoxification was not carried out in the case of opioids, but at best a dose reduction was undertaken and substitution treatment continued. Cannabis withdrawal treatment did not take place as part of the inpatient treatment, so it can be assumed that patients continued to use cannabis after discharge.

### Acquisition of demographic data

Demographic characteristics—i.e., age, sex, and medical diagnoses—were obtained from patient records.

### Medication evaluation tools

Drug prescriptions were analyzed by an interdisciplinary expert panel of specialists in psychiatry, neurology and clinical pharmacology. To evaluate prescription patterns of different drug classes, drugs were categorized according to the World Health Organization’s Anatomical Therapeutic Chemical (ATC) classification system.

Moreover, the drugs.com classification (Drugsite Trust, Auckland, New Zealand) and the electronic drug interaction program UpToDate Lexicomp^®^ (Wolters Kluwer Clinical Drug Information) were utilized for the evaluation of potential CDIs and DDIs.

Drugs.com provides information on possible interactions between different drugs and psycho-tropic substances including cannabis. It contains data on the severity of the interaction, possible ADRs and precautions to take. The database covers information on 393 drugs possibly involved in CDIs. Thereby, 27 possible CDIs are classified as major and 366 as moderate.

Patients’ medication charts were screened for DDIs using the electronic drug interaction program UpToDate Lexicomp^®^. Only DDIs classified as ‘avoid combination’, or ‘consider therapy modification’ by UpToDate Lexicomp^®^ were included in the statistical analysis.

### Statistics

Microsoft^®^ Excel^®^ 2019 (Redmond, Washington, USA) and IBM^®^ SPSS^®^ Statistics 28 (Armonk, New York, USA) were used for statistical analysis. Descriptive statistical methods were used to summarize the data. Continuous variables are depicted as means ± standard deviations (SDs) or as medians with interquartile ranges (IQRs). For categorical variables, absolute and relative frequencies were calculated. For quantitative variables, the Mann-Whitney *U* test was used to investigate potential differences between groups. Statistical significance was defined as a two-sided *p*-value = < 0.05.

## Results

### Study population and medication

In this study, we manually screened a total of 301 patient cases involving 179 individual patients for potential CDIs and DDIs. The discrepancy between the number of patient cases and individual patients is due to the inclusion of returning patients. Median age of the patient cohort was 37 years (interquartile range (IQR) 32–43 years; range 18–57 years), with a predominance of male sex (85.0%; 256/301). Patients were prescribed a median of 3 drugs (IQR 1–4; range 0–15). No statistically significant differences could be detected between men and women regarding the number of taken drugs (*p* = 0.786). Polypharmacy, defined as the simultaneous use of five or more different drugs, was observed in 21.9% (66/301) of patients. The patient cohort consisted only of recreational cannabis users.

Table 1 provides a detailed overview of psychiatric diagnoses and somatic comorbidities in the study population. Apart from CUD, alcohol use disorder (AUD), present in 70.4% (212/301) of patient cases, and cocaine use disorder (48.4%; 147/301) as well as depression (34.9%; 105/301) were the most prevalent psychiatric diagnoses. Arterial hypertension was the most common somatic comorbidity, affecting 5.0% (15/301) of the study population. A total of 992 drugs, comprising 153 different substances, were prescribed to the study cohort. The three most commonly prescribed drugs were levomethadone (8.4%; 83/992), pantoprazole (7.2%; 71/992) and levetiracetam (5.2%; 52/992).

**Table 1:** Characteristics of the study population (*n* = 301). The median age of the study cohort was 37 years (IQR = 32–43)

Variables	*n*	%
**Sex**		
Female	45	15.0
Male	256	85.0
**Psychiatric diagnoses** ^**a**^		
Cannabis use disorder	301	100.0
Multiple substance use disorder	29	9.6
Sedative use disorder	75	24.9
Alcohol use disorder	212	70.4
Cocaine use disorder	144	47.8
Opioid use disorder	147	48.8
Amphetamine use disorder	41	13.6
Depression	105	34.9
Bipolar affective disorder	3	1.0
Schizophrenia or schizophreniform disorder	21	7.0
Personality disorder	77	25.6
PTSD	21	7.0
Delirium	3	1.0
Other psychiatric disorder(s)	140	46.5
**Somatic diagnoses** ^**a**^		
Arterial hypertension	15	5.0
Coronary heart disease	3	1.0
Chronic heart failure	4	1.3
Status post stroke	7	2.3
Type-2 diabetes mellitus	12	4.0
Chronic obstructive pulmonary disease	3	1.0
Hypothyroidism	8	2.7
Urinary tract infection	4	1.3
Epilepsy	12	4.0
Other somatic disorder(s)	181	60.1

^a^Patients could have more than one diagnosis

**Table 2:** Prevalence of the ATC categories of drugs involved in potential cannabis-drug interactions according to drugs.com (*n* = 543)

ATC-Classification	*n*	%
All pCDIs	543	100
Severe pCDIs	124	100
N07 OTHER NERVOUS SYSTEM DRUGS	117	94.4
N02 ANALGESICS	7	5.6
**Moderate pCDIs**	**419**	**100**
N05 PSYCHOLEPTICS	145	34.6
N06 PSYCHOANALEPTICS	120	28.6
N03 ANTIEPILEPTICS	61	14.6
C09 AGENTS ACTING ON THE RENIN-ANGIOTENSIN SYSTEM	32	7.6
N02 ANALGESICS	31	7.4
C07 BETA BLOCKING AGENTS	10	2.4
C03 DIURETICS	8	1.9
C08 CALCIUM CHANNEL BLOCKERS	6	1.4
A07 ANTIDIARRHEALS, INTESTINAL ANTI-INFLAMMATORY/ANTI-INFECTIVE AGENTS	1	0.2
C02 ANTIHYPERTENSIVES	1	0.2
J01 ANTIBACTERIALS FOR SYSTEMIC USE	1	0.2
N01 ANESTHETICS	1	0.2

**Table 3:** Prevalence of the ATC categories of drugs involved in potential drug-drug interactions according to UpToDate Lexicomp^®^ (*n* = 392)

ATC-Classification	*n*	%
All drugs involved into pDDIs	392	100
Avoid combination	100	100
N05 PSYCHOLEPTICS	67	67.0
N07 OTHER NERVOUS SYSTEM DRUGS	27	27.0
N02 ANALGESICS	2	2.0
J05 ANTIVIRALS FOR SYSTEMIC USE	1	1.0
L01 ANTINEOPLASTIC AGENTS	1	1.0
N03 ANTIEPILEPTICS	1	1.0
N06 PSYCHOANALEPTICS	1	1.0
**Consider therapy modification**	**292**	**100**
N05 PSYCHOLEPTICS	105	36.0
N07 OTHER NERVOUS SYSTEM DRUGS	68	23.3
N06 PSYCHOANALEPTICS	53	18.2
N02 ANALGESICS	31	10.6
N03 ANTIEPILEPTICS	24	8.2
J05 ANTIVIRALS FOR SYSTEMIC USE	6	2.1
R03 DRUGS FOR OBSTRUCTIVE AIRWAY DISEASES	2	0.7
L01 ANTINEOPLASTIC AGENTS	1	0.3
M01 ANTIINFLAMMATORY AND ANTIRHEUMATIC PRODUCTS	1	0.3
R06 ANTIHISTAMINES FOR SYSTEMIC USE	1	0.3

### Potential cannabis-drug interactions

With the aid of the drugs.com classification, 54.7% (543/992) of all prescribed drugs were identified as potentially interacting with cannabis (Table 2). 89.4% (269/301) of all patient cases involved were affected by the prescription of at least one drug that could potentially interact with cannabis. No statistically significant differences were found between men and women with regard to the number of potential CDIs (*p* = 0.155).

12.5% of all prescribed drugs (124/992) were involved in CDIs with major interaction potential. The three most frequently prescribed drugs associated with these potentially major interactions were levomethadone (66.9%; 83/124), buprenorphine (21.0%; 26/124), and morphine (6.5%; 8/124) (Supplementary Table 2). Additionally, 42.2% (419/992) of all prescribed drugs were afflicted with potentially moderate CDIs. Among these, the three most commonly prescribed drugs were levetiracetam (12.4%; 52/419), quetiapine (9.3%; 39/419), and mirtazapine (8.8%; 37/419) (Supplementary Table 2).

The two drug classes most frequently associated with potentially severe CDIs were “Other nervous system drugs” (N07) (94.4%; 117/124) and “Analgesics” (N02) (5.6%; 7/124). Regarding potentially moderate CDIs, the three most commonly involved drug classes were “Psycholeptics” (N05) (34.6%; 145/419), “Psychoanaleptics” (N06) (28.6%; 120/419), and “Antiepileptics” (N03) (14.6%; 61/419).

### Potential drug-drug interactions

In total, 196 DDIs could be detected. Of these, 25.5% (50/196) were categorized as “avoid combination”, while 74.5% (146/196) were categorized as “consider therapy modification” (Table 3). No statistically significant differences were found between men and women with regard to the number of potential DDIs (*p* = 0.525). 40.2% (121/301) of all patient cases involved were affected by the prescription of at least one potential DDI.

Drugs most frequently involved in DDIs categorized as “avoid combination” were levomethadone (27.0%; 27/100), quetiapine (26.0%; 26/100), and buprenorphine (21.0%; 21/100). Herein, the combinations of levomethadone and quetiapine (26.0%; 13/50) and levomethadone and flupentixol (10.0%; 8/50) were commonly identified. For DDIs categorized as “consider therapy modification,” the most frequently affected drugs were levomethadone (23.3%; 68/292), pipamperone (8.2%; 24/292), and buprenorphine (7.2%; 21/292). Hereby, the most prevalent combinations were levomethadone and diazepam (7.5%; 11/146) and levomethadone and oxazepam (6.8%; 10/146) as well.

Among the “avoid combination” category, the most frequently affected ATC groups were psycholeptics (N05) (67.0%; 67/100), other nervous system drugs (N07) (27.0%; 27/100), and analgesics (N02) (2.0%; 2/100). In the “consider therapy modification” category, ATC groups commonly involved in potential DDIs were psycholeptics (N05) (36.0%; 105/292), other nervous system drugs (N07) (23.3%; 68/292), and psychoanaleptics (N06) (18.2%: 53/292).

## Discussion

The present study investigated the prevalence and characteristics of potential CDIs and DDIs in a sample of inpatients during treatment on an addiction-specific ward of a university hospital in Germany over a period of six years. Two different tools to detect potential drug interactions were used, namely the drugs.com classification for CDIs and UpToDate Lexicomp^®^ for the detection of DDIs. To the best of our knowledge, this is the first study to apply these tools for the detection of potential drug interactions in patients with CUD.

The mean age of our study population was approximately 36 years, and the most common psychiatric diagnoses beside CUD were also substance use disorders followed by depression. Thereby, our study collective showed great similarities with foregoing studies on inpatients with CUD in terms of age, sex and comorbidity burden (Ricci et al. 2021; Oladunjoye et al. 2022).

There are now several case reports and some reviews in the literature that illustrate potential clinical consequences of interactions between cannabinoids and individual drugs (Geffrey et al. 2015; Grayson et al. 2018; Nasrin et al. 2021). Evidence has been generated that the combination of cannabis use with intake of the anticoagulant drug warfarin increases the risk of bleeding complications (Damkier et al. 2019). The exact mechanisms behind this are partially unclear, although the results of a study by Bansal et al. indicate that CBD inhibits CYP2C19, but the previously postulated inhibition of CYP2C9 by THC was not verified (Bansal et al. 2023). Accordingly, the probability of interactions between cannabis and warfarin was classified as very high in a systematic review (Lopera et al. 2022). A similar level of evidence is also available for clobazam, whereby CBD can lead to the accumulation of clobazam via inhibition of CYP3A4 and CYP2C19 (Geffrey et al. 2015). However, several studies have yet investigated potentially beneficial effects of this pharmacokinetic interaction for the treatment of rare forms of epilepsy (Geffrey et al. 2015; Golub and Reddy 2021). A risk of pharmacokinetic and pharmacodynamic interactions with cannabis can also be stated for several other drugs (Lopera et al. 2022). Two recent systematic reviews of Nachnani et al. and Maldonado an Colleagues have proven that interactions of cannabinoids with concomitantly prescribed drugs are likely and the strongest evidence has been generated for warfarin, valproate, tacrolimus, and sirolimus (Maldonado et al. 2024; Nachnani et al. 2024). An evaluation which of these drugs are prescribed particularly frequently to patients with CUD is not available yet.

Within our study, the majority of patient cases was affected by potential CDIs. The most frequently prescribed drugs associated with major risk of CDIs were levomethadone, buprenorphine, and morphine. Levomethadone and buprenorphine are utilized for substitution treatment of patients with opioid use disorder, which illustrates the risk of potential CDIs in this patient group in particular.

A study conducted by Vierke et al. indicates that cannabis consumption leads to a reduction in the formation of norbuprenorphine and an elevation in the levels of buprenorphine and norbuprenorphine in the blood, likely due to the inhibition of CYP3A4 enzyme (Vierke et al. 2021). This pharmacokinetic interaction could potentially lead to heightened or modified opioid effects and an increased risk of intoxication (Vierke et al. 2021). Comparable pharmacokinetic interaction potentials have also been shown for levomethadone and methadone. This should result in clinical monitoring for opioid intoxication symptoms in patients with CUD and concomitantly prescribed opioid substitution therapy. On the other hand, positive effects of such interactions can also be utilized. So, Abrams et al. found that vaporized cannabis given to patients with chronic pain on opioid therapy (morphine or oxycodone) increased the analgesic effect of opioids (Abrams et al. 2020).

Among potentially moderate CDIs, the three most affected drugs were levetiracetam, quetiapine, and mirtazapine. The interaction potential of cannabinoids and antiepileptic drugs has repeatedly been characterized in the literature, although data on levetiracetam are sparse (Lucas et al. 2018). Hereby, a mouse study identified that CBD decreased antiseizure activity of levetiracetam against externally induced psychomotor seizures (Socała et al. 2019). Possible CDIs involving quetiapine and mirtazapine are also due to the influence on metabolization via CYP isoenzymes resulting in an increase of sedation and psychomotor slowing (Lucas et al. 2018). In general, drugs with influence on the central nervous system (CNS) were commonly involved in potential CDIs in our study population. This in turn suggests that vigilance should be monitored in patients with CUD, and that the indication for sleep-inducing and sedative drugs should be given rather cautiously.

Patients with CUD are often affected by polypharmacy due to their comorbidity profile and are therefore particularly susceptible to the development of ADRs caused by possible DDIs (Connor et al. 2021). However, a systematic recording of potential DDIs in patients with CUD has so far only been carried out for other addiction disorders, but not for CUD (Guerzoni et al. 2018; Schröder et al. 2024). In the context of AUD, benzodiazepines and disulfiram have been shown to be frequently involved in potential DDIs (Guerzoni et al. 2018). A retrospective cohort study by Schröder et al. also identified the combination of potassium supplements with renin-angiotensin-aldosterone system inhibitors to be commonly responsible for potentially severe DDIs in geriatric patients with AUD (Schröder et al. 2024).

The results of the present study have shown that DDIs are also a common phenomenon in patients with CUD. Buprenorphine and levomethadone were particularly frequently involved in the 196 potential DDIs in our collective, both in the category of combinations to be avoided and those to be critically evaluated. These drugs for opioid substitution treatment exhibited extensive interaction potential with other drugs that influence CNS functions (such as benzodiazepines and antipsychotic drugs). Therefore, these DDIs can also result in a potentiation of CNS depressive effects, analogous to the CDIs outlined above. This in turn possibly leads to states of confusion, risk of falls and reduced vigilance. Furthermore, most of the drugs involved in potential DDIs in our study bear the risk of prolongation of the QT interval (Sarganas et al. 2014). This should lead to electrocardiographic (ECG) controls in this patient population to prevent it from malignant cardiac arrythmias.

In summary, the results of the present study reveal that potential CMIs and DDIs are common among patients with CUD. The interaction potential of cannabinoids is especially due to their influence on the activity of CYP isoenzymes, which should be considered when prescribing drugs metabolized via this pathway in patients with CUD. In addition, drugs used for opioid substitution treatment were often involved in potential CMIs and DDIs. The interaction of levomethadone or buprenorphine with cannabinoids and other drugs with effects on CNS functions can in turn result in CNS depressive effects, whereby their occurrence should be clinically monitored. So, especially patients suffering from opioid use disorder in addition to CUD seem at risk for potential drug interactions and consecutive ADRs. The findings of our study indicate that a significant portion of drugs prescribed to patients with CUD should be critically evaluated in accordance with the drugs.com list and the UpToDate Lexicomp^®^ interaction check. UpToDate Lexicomp^®^ was used because of its easy availability and thus the potentially good reproducibility of our results and associated possibilities for implementation in clinical routine. On the other hand, the utilized interaction tools showed good performance in foregoing studies with regard to the detection of potentially clinically relevant drug interactions (Muhič et al. 2017; Marcath et al. 2018). However, it is important to note that none of the applied tools was specifically designed for addiction psychiatry but rather for assessing drug safety in patients with CUD. Therefore, a comprehensive assessment of prescribed drugs in patients with CUD requires thorough analysis of the benefits and risks, as well as careful consideration of alternative pharmacological options. Nevertheless, the results of our study should have some more implications for clinicians regarding the care of patients with CUD. As patients with CUD are particularly affected by CDIs and DDIs, withdrawal treatment from cannabis or other psychotropic substances should be done with caution in this patient group and should contain close monitoring of respiratory and cardiac functions as well as repeated measures of drug serum concetrations. Furthermore, since drugs used for opioid substitution treatment were frequently involved in CDIs and DDIs, complete withdrawal from opioids should be discussed in patients with opioid use disorder and comorbid CUD. In order to quantify effects of possible drug interactions, methods such as therapeutic drug monitoring (TDM) should be incorporated even more into clinical routine (Hiemke et al. 2018).

As limitations, the applied drug interaction tools do not specify pharmacological alternatives. Furthermore, our study cohort only contained recreational cannabis consumers, so no conclusions can be drawn regarding drug interactions with medicinal cannabis. Moreover, the monocentric design and its exclusive setting within a specialized unit of a university hospital has to be mentioned. Consequently, the generalizability of our findings to other healthcare settings may be limited. In addition, the retrospective nature of our study prevents us from determining whether the identified potential CDIs and DDIs resulted in adverse outcomes in our population. To overcome these limitations, future research should use a prospective design to thoroughly analyze risks of adverse outcomes associated with drug interactions in patients with CUD. Serum levels of the drugs potentially interacting with cannabis could then also be determined to enable a more detailed description of the clinical relevance of CDIs and their course during withdrawal treatment. Such studies will allow healthcare professionals to accurately stratify individuals with CUD based on their risk profile at the time of prescribing. In addition, randomized controlled trials should be conducted to prospectively assess whether addressing potential CDIs and DDIs can reduce the incidence of adverse effects in patients with CUD.

## Electronic supplementary material

Below is the link to the electronic supplementary material.

Supplementary material 1

## Data Availability

The data that support the findings of this study are available upon reasonable request from the corresponding author.

## Abbreviations

ADR: Adverse drug reaction
AUD: Alcohol use disorder
CBD: Cannabidiol
CDI: Cannabis-drug interaction
CNS: Central nervous system
CUD: Cannabis use disorder
CYP: Cytochrome P450
DDI: Drug-drug interaction
DSM-5: Diagnostic and Statistical Manual of Mental Disorders 5
ICD-10: International Classification of Diseases-10
IQR: Interquartile range
TDM: Therapeutic drug monitoring
THC: Delta-9-tetrahydrocannabinol
UGT: UDP-glucuronosyltransferase
