# Supplementary material for: Drug interactions in a sample of inpatients diagnosed with cannabis use disorder
Source: J Neural Transm (Vienna). 2025 Jan 23;132(5):723–30. doi: 10.1007/s00702-025-02884-5 (PMC12043780; doi:10.1007/s00702-025-02884-5)
Supplement: Supplementary file 1 — Supplementary material 1 [file 702_2025_2884_MOESM1_ESM.docx]

**SUPPLEMENTARY TABLE 1** All drugs involved in pCDIs in the study population (n = 543)

| **ATC-Classification** | **n** | **%** |
| --- | --- | --- |
| **All pCDIs** | **543** | **100** |
| **Severe pCDIs** | **124** | **100** |
| Levomethadone | 83 | 66.9 |
| Buprenorphine | 26 | 21.0 |
| Morphine | 8 | 6.5 |
| Tramadol | 3 | 2.4 |
| Hydromorphone | 2 | 1.6 |
| Oxycodone | 2 | 1.6 |
| **Moderate pCDIs** | **419** | **100** |
| Levetiracetam | 52 | 12.4 |
| Quetiapine | 39 | 9.3 |
| Mirtazapine | 37 | 8.8 |
| Sertraline | 33 | 7.9 |
| Pregabalin | 31 | 7.4 |
| Ramipril | 31 | 7.4 |
| Oxazepam | 24 | 5.7 |
| Risperidone | 24 | 5.7 |
| Aripiprazole | 20 | 4.8 |
| Doxepin | 18 | 4.3 |
| Venlafaxine | 14 | 3.3 |
| Diazepam | 11 | 2.6 |
| Olanzapine | 11 | 2.6 |
| Bisoprolol | 7 | 1.7 |
| Haloperidol | 7 | 1.7 |
| Amlodipine | 5 | 1.2 |
| Promethazine | 5 | 1.2 |
| Valproate | 4 | 1.0 |
| Amitriptyline | 3 | 0.7 |
| Carbamazepine | 3 | 0.7 |
| Duloxetine | 3 | 0.7 |
| Fluoxetine | 3 | 0.7 |
| Hydrochlorothiazide | 3 | 0.7 |
| Paroxetine | 3 | 0.7 |
| Torasemide | 3 | 0.7 |
| Trimipramine | 3 | 0.7 |
| Cannabidiol | 2 | 0.5 |
| Escitalopram | 2 | 0.5 |
| Furosemide | 2 | 0.5 |
| Lithium | 2 | 0.5 |
| Metoprolol | 2 | 0.5 |
| Cariprazine | 1 | 0.2 |
| Carvedilol | 1 | 0.2 |
| Ciprofloxacin | 1 | 0.2 |
| Citalopram | 1 | 0.2 |
| Clonazepam | 1 | 0.2 |
| Clozapine | 1 | 0.2 |
| Doxazosin | 1 | 0.2 |
| Enalapril | 1 | 0.2 |
| Lamotrigine | 1 | 0.2 |
| Levocetirizine | 1 | 0.2 |
| Lidocaine | 1 | 0.2 |
| Verapamil | 1 | 0.2 |

**SUPPLEMENTARY TABLE 2** All drugs involved in pDDIs in the study population (n = 392)

| **Drug** | **n** | **%** |
| --- | --- | --- |
| **All prescribed medications** | **392** | **100** |
| **Avoid combination** | **100** | **100** |
| Levomethadone | 27 | 27.0 |
| Quetiapine | 26 | 26.0 |
| Pipamperone | 21 | 21.0 |
| Flupentixol | 9 | 9.0 |
| Risperidone | 4 | 4.0 |
| Haloperidol | 3 | 3.0 |
| Olanzapine | 3 | 3.0 |
| Citalopram | 1 | 1.0 |
| Carbamazepine | 1 | 1.0 |
| Efavirenz | 1 | 1.0 |
| Buprenorphine | 1 | 1.0 |
| Lenvatinib | 1 | 1.0 |
| Oxazepam | 1 | 1.0 |
| Metamizole | 1 | 1.0 |
| **Consider therapy modification** | **292** | **100** |
| Levomethadone | 68 | 23.3 |
| Pipamperone | 24 | 8.2 |
| Buprenorphine | 21 | 7.2 |
| Mirtazapine | 16 | 5.5 |
| Sertraline | 15 | 5.1 |
| Diazepam | 14 | 4.8 |
| Doxepin | 14 | 4.8 |
| Quetiapine | 13 | 4.5 |
| Levetiracetam | 12 | 4.1 |
| Aripiprazole | 12 | 4.1 |
| Oxazepam | 12 | 4.1 |
| Pregabalin | 10 | 3.4 |
| Flupentixol | 9 | 3.1 |
| Haloperidol | 6 | 2.1 |
| Levomepromazine | 6 | 2.1 |
| Efavirenz | 5 | 1.7 |
| Olanzapine | 4 | 1.4 |
| Risperidone | 4 | 1.4 |
| Amitriptyline | 3 | 1.0 |
| Morphine | 3 | 1.0 |
| Tramadol | 3 | 1.0 |
| Donepezil | 2 | 0.7 |
| Salbutamol | 2 | 0.7 |
| Oxycodone | 2 | 0.7 |
| Venlafaxine | 2 | 0.7 |
| Carbamazepine | 1 | 0.3 |
| Amisulpride | 1 | 0.3 |
| Ibuprofen | 1 | 0.3 |
| Dolutegravir | 1 | 0.3 |
| Levocetirizine | 1 | 0.3 |
| Lenvatinib | 1 | 0.3 |
| Metamizole | 1 | 0.3 |
| Clonazepam | 1 | 0.3 |
| Atomoxetine | 1 | 0.3 |
| Hydromorphone | 1 | 0.3 |
